# Domestic Medicine

**Published:** 1870-07

**Authors:** 


### Domestic Medicine.

HOW TO TAKE QUININE.—The bitter taste of quinine is completely concealed by taking it in sweet milk.

---

TO REDUCE A HERNIA OR RUPTURE.—A hernia can almost invariably be reduced, by laying the patient upon his back and giving him a cup of strong coffee to drink, every fifteen or twenty minutes. The coffee must be taken clear, i. e. without milk or sugar, and must be pure and not adulterated stuff.

The reduction is the result of the contraction of the muscular fibres of the intestines, by the stimulating action of the coffee.

---

QUINSY.—Persons who are subjected to this disease of the tonsels, will be glad to learn that there is a process by which it can be arrested.

The moment the soreness about the throat is discovered, saturate a flannel cloth with common kerosene oil, place it about the throat, well up under the ears, and confine it there with a towel wound about the neck. Keep the application there all night, or as long as the soreness *inside* continues. The remedy will cause considerable redness and perhaps slight blistering of the skin, but will as surely arrest the disease and prevent suppuration of the gland. We have seen the remedy tried in numerous instances, and know that it will arrest quinsy.

---

TO ARREST BLEEDING.—Having recently seen a fatal case of bleeding, in a young man who cut his foot, while in the woods lumbering, and who was allowed to bleed to death in the presence of his companions in consequence of their ignorance with reference to the management of an injury of this character, we have concluded that the means by which hemorrhage can be at once arrested, is not generally understood.

When the hands, feet, arms or legs are the injured portions of the body, the flow of blood can be instantly stopped by tying a handkerchief or piece of rope, loosely about the arm or leg, *above* the point of injury; then, by thrusting a stick under the handkerchief and next the skin, commence gradually to twist the ligature by turning the stick, until it is made sufficiently tight to arrest all bleeding.

Never try to stop the blood by stuffing the wound with lint or rags. Such a process is worse than useless. If the cut is about the trunk of the person, the wound may be plugged with cotton or lint, and firm pressure made upon the wound with the hand, until the surgeon arrives. If in the winter time, a large snow-ball should be pressed against the wound while the messenger summons the physician.

---

**WORMS IN CHILDREN.**—If you have reason to suspect worms in the intestines of your children, as is evinced by the ordinary symptoms of colic-pains, hardness of the abdomen, flow of saliva, itching of the nose, offensive breath, disturbed sleep, and impaired appetite, you can free them of their unwelcome tenants by the administration of a dose that the little ones will not refuse to take. Procure an ounce of the "Fluid Extract of Pinkroot and Senna," and give, to a child from 2 to 5 years of age, a dessert spoonful every morning, before eating, until you have reason to believe that all the worms have been expelled.

The medicine is sweet to the taste and will be readily taken by the child. We have never known it to fail in destroying the worms.

---

**DYSPEPSIA.**—If you are troubled with a coated tongue, and bad taste in your mouth in the morning, an offensive breath, a sense of weight in your stomach, and a sort of stricture in your throat as though your food had lodged there, your difficulty is indigestion—your food decomposes or rots in your stomach, instead of undergoing a thorough digestion. This can be remedied, partly by regulating your diet and partly by proper medicine. You must eat less; give your stomach an opportunity to rest; you have over-taxed it by gluttony or by eating at unseasonable hours. If you have been in the habit of eating three hearty meals a day, and are leading a sedentary life, you are eating much more than your system requires—you are over-taxing your stomach. Then, if you sometimes eat fried oysters, pig's feet and other luxuries, drink liquors and smoke cigars late at night, at a party, or with friends at home, you are requiring more of your stomach than you have a right to demand. It will rebel—the juices necessary for healthy digestion will cease to flow, and you will fill your stomach with another meal before it has disposed of its last one; you will awake in the morning with the symptoms first described, and in a short time will be that American biped—a dyspeptic.

The cure is easy:—Take but one meal a day for a week or two. Eat in the middle of the day. Let your meal consist of a broiled tenderloin-steak, with no dressing save its own juice. Avoid butter, pepper and salt, upon your meat. You will be surprised to learn how much better the flavor of a steak is without the melted butter that cooks are wont to souse it with. One baked potato, a little stewed tomato, some brown-bread and a glass of Scotch ale, is enough for any body's dinner. If you are doing an office or store business, you can live very easily on this for one week. Then, after so resting your stomach, take a teaspoonful of the following tonic every morning, before breakfast:

Take,
Tincture chloride of iron, one oz;
Quinine, twenty grains;
Water, eight ounces.
Mix.

After which, eat a piece of toast moistened with milk, with a poached egg upon it, and drink a cup of black tea. This is sufficient for breakfast. For dinner eat your steak, potato, &c., as before; and for supper eat nothing but a bowl of boiled, cracked-wheat and milk. If you will follow up this treatment of your stomach for three months, you will succeed in curing a very troublesome disease; and, if ever afterward you will avoid late suppers, smoking, strong drink and immoderate eating, you will never have occasion to again call yourself a dyspeptic.

---

"**BOWEL COMPLAINT.**"—During the hot weather both children and grown folks are liable to derrangements of the bowels. The little ones produce a slight diarrhœa from a too free indulgence in berries and fruit, while the working portion of the people bring on the same trouble from excessive drinking and hard work in the hot sun.

The following mixture should be prepared and always kept in the house. It will be found useful in all manner of "bowel complaints," or for pain in stomach or bowels. Take of—

Laudanum,

Tincture of Camphor,

Tincture ot Capsicum.

Of each two (2) drachms. Mix, and give a child 2 years old, from 3 to 5 drops, on sugar, three times a day. For an adult half a teaspoonful may be taken, in same manner, for one dose.

---

"**SUN STROKE.**"—A man who has fallen from sun-stroke should be ministered to at once. Too much valuable time is consumed in attempting to carry him to his home before anything is done for his relief. Carry him to the shade of some tree or fence, lay him upon his back, on the ground ; fold his coat or one of some by-stander, and place it under his shoulders.—Open his shirt and expose his chest and throat freely to the air. Rub his breast, arms and legs in whisky or alcohol, and dash cold water upon his head. While this is being done send for ice, and when it arrives, pound it fine, place it in a salt sack or between the folds of a pocket handkerchief, and apply to his head and the nape of his neck. As soon as he can swallow, give him a little brandy and water. You can soon restore him to consciousness, when he can be removed to his residence and placed under the care of his physician.

